# 287. Characteristics and Outcomes of COVID-19 Patients with Candidemia at a Community Hospital in Chicago

**DOI:** 10.1093/ofid/ofab466.489

**Published:** 2021-12-04

**Authors:** Oluwadamilola A Adeyemi, Gregg Gonzaga, Sean Cariño, Steve B Kalish

**Affiliations:** 1 Swedish Hospital Part of NorthShore University HealthSystem, Chicago, Illinois; 2 Swedish Hospital Part of Northshore University HealthSystem, Chicago, Illinois

## Abstract

**Background:**

1,416 patients with acute COVID-19 infection were admitted to our hospital in 2020. During that year we noticed an alarming increase in cases of nosocomial Candidemia: 26 versus an average of 2.8 cases per year over the previous 5 years. 19 of the 26 episodes (73%) of Candidemia occurred in patients who were admitted with acute COVID-19 infection. Recent reports suggest that hospitalized patients with COVID-19 are at increased risk for developing Candidemia, however their clinical characteristics, risk factors and outcomes have not been well described. We evaluated the risk factors and mortality of hospitalized COVID-19 patients with Candidemia.

**Methods:**

We performed a retrospective chart review of 19 patients with Candidemia and confirmed COVID-19 infection at a 292-bed community teaching hospital in Chicago, Illinois from January through December 2020. We report a descriptive analysis of the demographic characteristics, comorbidities, complications, and outcomes of these patients.

**Results:**

The average age of our study population was 65 years; 68% were male. The average hospital length of stay (LOS) was 34 days. The mean time from admission to the development of Candidemia was 16 days. Associated co-morbidities included cardiovascular diseases (CVD) in 79%, diabetes mellitus (DM), in 68%, and obesity in 50%. Underlying kidney disease was present in 10%. Treatments for COVID-19 included convalescent plasma (53%), remdesivir (53%), steroids (52%) and tocilizumab (19%). All patients were managed in the intensive care unit (ICU) and 95% required multiple central line (CL) placements. Most of the patients (58%) required hemodialysis (HD); all patients were treated with multiple antibiotics. The average LOS in the ICU was 25 days. Despite anti-fungal treatment, 68% expired. The 28-day mortality was 50%.

**Conclusion:**

The occurrence of Candidemia in our hospitalized patients with acute COVID-19 infection was associated with a history of CVD, DM, obesity, prolonged hospital LOS, requirement for multiple CL, HD, treatment with multiple antibiotics and a long stay in the ICU. The mortality of COVID-19 patients with Candidemia is high. The development of strategies to mitigate the occurrence of nosocomial Candidemia in this population of patients is urgently needed.

**Disclosures:**

**All Authors**: No reported disclosures

